# The potential promise and pitfalls of point-of-care viral load monitoring to expedite HIV treatment decision-making in rural Uganda: a qualitative study

**DOI:** 10.1186/s12913-024-11747-w

**Published:** 2024-10-22

**Authors:** Joseph G. Rosen, William G. Ddaaki, Neema Nakyanjo, Larry W. Chang, Anh Van Vo, Tongying Zhao, Gertrude Nakigozi, Fred Nalugoda, Godfrey Kigozi, Joseph Kagaayi, Thomas C. Quinn, M. Kate Grabowski, Steven J. Reynolds, Caitlin E. Kennedy, Ronald M. Galiwango

**Affiliations:** 1 Department of International Health, Bloomberg School of Public Health, Johns Hopkins University, 615 N. Wolfe Street, Room E5031, Baltimore, MD 21205 USA; 2 Rakai Health Sciences Program, Entebbe, Uganda; 3 Division of Infectious Diseases, School of Medicine, Johns Hopkins University, Baltimore, MD USA; 4 Division of Intramural Research, National Institute of Allergy and Infectious Diseases, National Institutes of Health, Bethesda, MD USA; 5 Department of Pathology, School of Medicine, Johns Hopkins University, Baltimore, MD USA

**Keywords:** Point-of-care testing, Routine viral load monitoring, HIV care, Antiretroviral therapy, Implementation science, Sub-saharan Africa

## Abstract

**Background:**

HIV treatment programs in Africa have implemented centralized testing for routine viral load monitoring (VLM), which may result in specimen processing delays inhibiting timely return of viral load results. Decentralized, point-of-care (PoC) VLM is a promising tool for expediting HIV clinical decision-making but remains unavailable in most African settings. We qualitatively explored the perceived feasibility and appropriateness of PoC VLM to address gaps along the viral load monitoring continuum in rural Uganda.

**Methods:**

Between May and September 2022, we conducted 15 in-depth interviews with HIV clinicians (facility in-charges, clinical officers, nurses, counselors) and six focus group discussions with 47 peer health workers from three south-central Ugandan districts. Topics explored centralized VLM implementation and opportunities/challenges to optimizing routine VLM implementation with PoC testing platforms. We explored perspectives on PoC VLM suitability and feasibility using iterative thematic analysis. Applying the Framework Method, we then mapped salient constraints and enablers of PoC VLM to constructs from the Consolidated Framework for Implementation Research.

**Results:**

Clinicians and peers alike emphasized centralized viral load monitoring’s resource-intensiveness and susceptibility to procedural/infrastructural bottlenecks (e.g., supply stockouts, testing backlogs, community tracing of clients with delayed VLM results), inhibiting timely clinical decision-making. Participants reacted enthusiastically to the prospect of PoC VLM, anticipating accelerated turnarounds in specimen processing, shorter and/or fewer client encounters with treatment services, and streamlined efficiencies in HIV care provision (including expedited VLM-driven clinical decision-making). Anticipated constraints to PoC VLM implementation included human resource requirements for processing large quantities of specimens (especially when machinery require repair), procurement and maintenance costs, training needs in the existing health workforce for operating point-of-care technology, and insufficient space in lower-tier health facilities to accommodate installation of new laboratory equipment.

**Conclusions:**

Anticipated implementation challenges, primarily clustering around resource requirements, did not diminish enthusiasm for PoC VLM monitoring among rural Ugandan clinicians and peer health workers, who perceived PoC platforms as potential solutions to existing inefficiencies within the centralized VLM ecosystem. Prioritizing PoC VLM rollout in facilities with available resources for optimal implementation (e.g., adequate physical and fiscal infrastructure, capacity to manage high specimen volumes) could help overcome anticipated barriers to decentralizing viral load monitoring.

## Background

Since 2013, the World Health Organization (WHO) has endorsed routine viral load monitoring (VLM) as the standard of care for persons living with HIV receiving antiretroviral therapy (ART) [1]. In Uganda, like other sub-Saharan African countries with generalized HIV epidemics, VLM is recommended twice in the first year of ART initiation (the first measurement six months after ART initiation), then once annually after 12 months on ART if viral load suppression is maintained [2]. Routine VLM verifies the continued effectiveness of prescribed ART regimens for people living with HIV and can identify emerging clinical complications, including treatment failure, earlier in the course of infection [3–5]. Effective implementation of routine VLM, thus, holds great promise in improving clinical outcomes for people living with HIV through earlier detection and response to HIV viremia.

To accommodate the high volume of dried bloods spot (DBS) and plasma specimens submitted for routine VLM, many countries have centralized viral load testing, using central laboratories and other district/regional hospitals (or “hubs”) to perform viral load quantification on specimens obtained from satellite HIV clinics [6]. Centralized VLM requires a robust workforce to operate high-throughput laboratory equipment and oversee costly specimen transport/processing logistics, which—if absent—pose challenges to implementation quality and sustainability [6]. Emerging evidence also indicates that adherence to VLM schedules and timely clinical response to viremia (e.g., ART regimen switch) remains suboptimal in under-resourced, overburdened health systems [7–9], suggesting the benefits of routine VLM for patients and providers have not been fully realized in some contexts. Alternatives to centralized VLM are, thus, needed most in settings with the highest HIV burdens and most constraints on resources required to sustain centralized systems.

Highly sensitive and specific point-of-care (PoC) viral load testing platforms [10] have emerged as promising technologies for circumventing the challenges of a centralized VLM system. PoC VLM platforms enable specimens to be collected, tested, and processed in a single healthcare encounter, potentially evading delays in returning VLM results and reducing costs associated with outsourcing VLM to reference laboratories [10]. The promise of viral load quantification proximity to facilities where specimens are collected also renders these technologies highly desirable to clinicians and patients alike [11–13]. Given their compactness, relative throughput potential, and user-friendliness [10], PoC VLM platforms may be well-suited for implementation in some adequately resourced HIV clinics with manageable client volumes in resource-constrained settings [14–16].

Despite their promise, PoC VLM testing platforms have been implemented almost exclusively for specific populations (e.g., adolescents, pregnant or breastfeeding persons) in sub-Saharan Africa [17–19], and their implementation outside of higher-resourced hospitals and specialty clinics has been more limited [20]. While most persons living with HIV in the region receive clinical services from outpatient clinics in the public sector [21], few of these facilities have access to PoC VLM technology [20]. In the limited lower-tier health facilities in sub-Saharan Africa where PoC VLM is available, its implementation has expedited viral load test result delivery to health providers and return to clients, enabling rapid clinical follow-up [20]. Despite the measurable benefits of VLM decentralization (e.g., timeliness of results return, rapid identification of clients experiencing virologic failure) [20, 22], many barriers to PoC VLM implementation persist, including human resource scarcities, unreliable infrastructure (e.g., unstable electricity and supply chain disruptions), and disparate or conflicting guidelines on client eligibility for PoC testing [12, 13].

Studies of the feasibility and appropriateness of PoC VLM in settings with high HIV burdens, where centralized VLM remains standard practice, are scarce. Accordingly, we conducted a qualitative study in rural Uganda with diverse representatives from the HIV clinical workforce, eliciting their perspectives of PoC VLM as a viable and suitable alternative to centralized VLM in satellite HIV clinics, or standalone facilities unattached to hospital systems offering more expansive specialty care services. Through in-depth interviews with HIV clinicians and focus group discussions with peer health workers, we uncovered anticipated enablers and constraints to PoC VLM implementation, which can helpfully inform future rollout and scale-up efforts in this rural setting.

## Methods

### Study setting

Data were collected as part of a larger qualitative study [23, 24] exploring longitudinal patterns and drivers of HIV service (dis)engagement, as well as strategies for enhancing linkage and return to HIV care, in three southern Uganda districts: Kyotera, Masaka, and Rakai. The study area is distinguished by a heterogenous HIV epidemic, with burdens varying widely (prevalence range: 9–43%) across rural agrarian communities, semi-urban trading centers, and fish landing sites along Lake Victoria [25]. Given its proximity to Lake Victoria and the Tanzanian international border, the study area is also characterized by high population mobility, including seasonal and periodic in-/out-migration [26–28].

HIV clinical services are primarily delivered through outpatient public sector health facilities, with support from the Uganda Ministry of Health and the U.S. President’s Emergency Plan for AIDS Relief. Uganda has a decentralized, tiered healthcare system, the foundation of which are health posts staffed by village health teams, equivalent to community health workers [29]. HIV care is available starting at the second-lowest tier of health facilities (i.e., level-II clinics). Uganda adopted a “Treat All” approach in December 2016, through which ART is offered to all clients at the time of HIV diagnosis, irrespective of CD4 cell count or clinical staging [30]. Except for pediatric populations and pregnant persons (whose viral load specimens are processed at district hubs), the Central Public Health Laboratory (CPHL) performs viral load quantification on the remaining DBS and plasma specimens collected from satellite HIV clinics across the country.

### Design and procedures

We began by mapping all registered health facilities providing HIV care and treatment services in the study area. Between May and September 2022, we approached facility in-charges at purposively selected facilities, seeking variation by facility level (i.e., level-II to regional referral hospital) and community type (i.e., rural agrarian, semi-urban trading, or lakeside fishing), and elicited permission to approach HIV clinicians and peer health workers (or “expert clients”) for study recruitment. Facility in-charges then nominated themselves or another HIV clinician (i.e., clinical officer, nurse, counselor) to screen for eligibility and enroll. Separately, we identified and recruited peer health workers, who are people living with HIV delivering auxiliary services at HIV clinics (e.g., social support, education, client tracing), across health facilities in the study area, purposively sampled by level/tier. We anticipated peer health workers could represent the perspectives of both persons living with HIV and the non-clinical health workforce supporting implementation of HIV care and treatment services in the study area.

Trained qualitative interviewers conducted semi-structured, in-depth interviews (~ 45–60 min) in English with HIV clinicians, exploring existing challenges to implementing centralized VLM in rural health clinics. Interviews also elicited provider perspectives on anticipated benefits and barriers to implementing PoC VLM in their respective facilities. Despite PoC VLM’s implementation at district hubs and referral hospitals at the time of recruitment, HIV clinicians exhibited strong familiarity with PoC testing platforms (i.e., for CD4 cell count monitoring) and were, thus, well-positioned to provide nuanced, albeit theoretical in most instances, commentary on the implementation determinants of PoC VLM. Separately, peer health workers participated in focus group discussions (~ 60–90 min), moderated by experienced facilitators in Luganda, the local language. Discussions similarly explored challenges implementing centralized VLM and potential opportunities for leveraging PoC testing to decentralize routine VLM. Clinicians and peer health workers received 20,000 Ugandan shillings (~ 5.50 USD) for their participation.

### Analysis

Audio-recorded interviews and focus groups were professionally transcribed and translated into English, when required. Guided by the Framework Method [31], we used multi-cycle, blended (inductive and deductive) thematic analysis [32, 33] to interrogate emerging clinician and peer health worker perspectives on PoC VLM’s feasibility and appropriateness in their practice contexts. We began through close (line-by-line) reading of transcripts, generating descriptive memos summarizing nascent themes related to centralized and PoC VLM. We transformed these nascent themes from the first analysis cycle into standalone initial codes. We further consolidated and refined these initial codes by mapping themes onto constructs articulated in the Consolidated Framework for Implementation Research (CFIR)—a deterministic conceptual model for prospectively identifying barriers and facilitators of implementation strategies for evidence-based interventions at multiple ecological levels [34].

After importing transcripts into ATLAS.ti version 9 (Scientific Software Development GmbH, Berlin, Germany), we applied CFIR-informed focused codes to all interview and focus group transcripts. We then exported, reviewed, and redisplayed coded text segments using data arrays [33], which facilitated identification of salient themes and relevant data patterns. We continued to refine salient thematic concepts and patterns through ongoing memo-writing, debriefing amongst the investigative team, and member checking (i.e., results dissemination to and facilitated dialogues with relevant stakeholders) [35].

## Results

We conducted 15 in-depth interviews with HIV clinicians (mean age: 36 years, 53% female, average duration of clinical practice: 8 years) across 15 health facilities, 60% of which were level-II or level-III facilities. Only two providers participated from facilities implementing PoC VLM at the time of recruitment. We also moderated six focus group discussions with 47 peer health workers (mean age: 42 years, 72% female) recruited from 12 facilities.

**Fig. 1:**
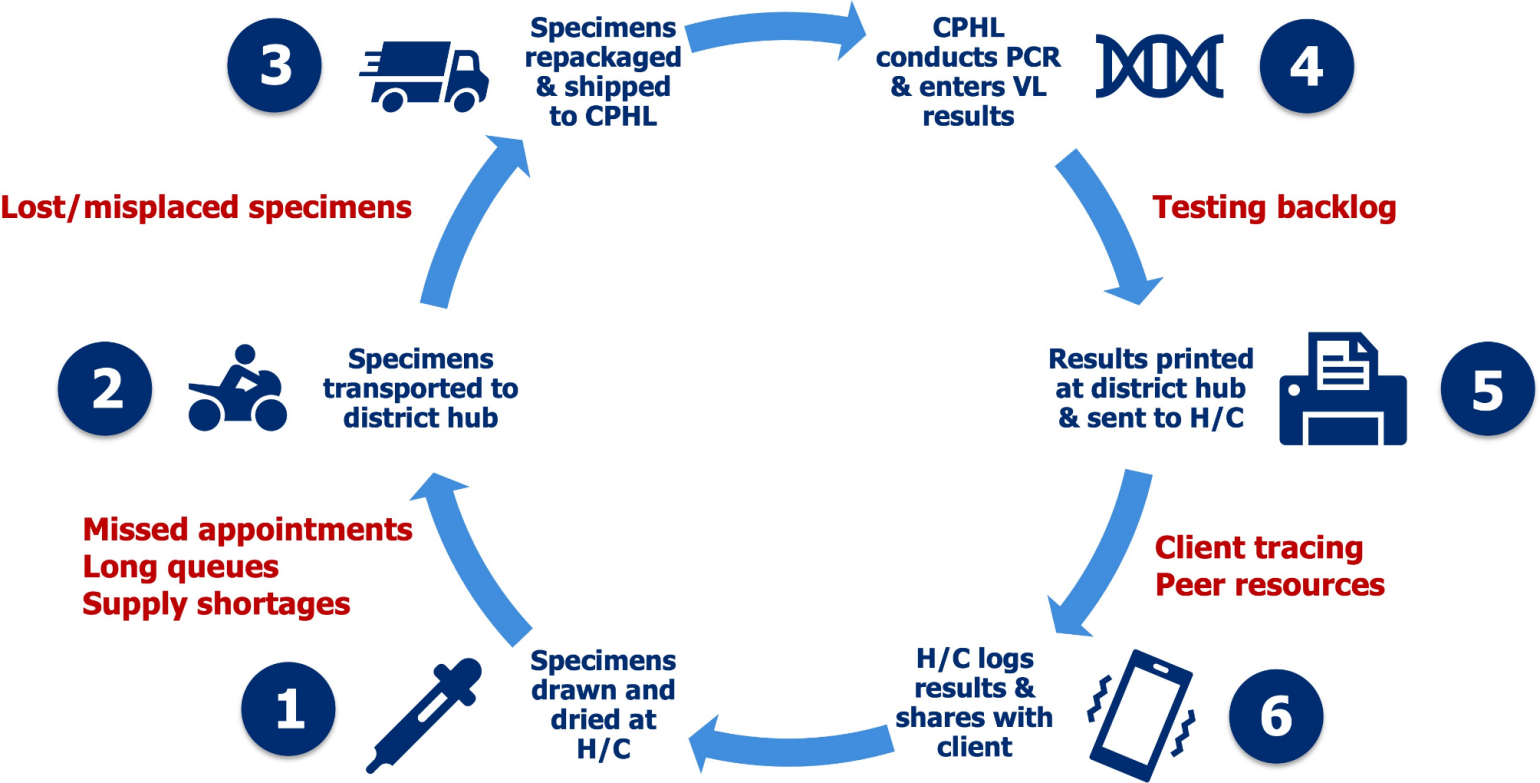
Provider-identified barriers and facilitators of point-of-care viral load Monitoring (PoC VLM), mapped to domains and constructs from the Consolidated Framework for Implementation. *Notes*: LTFU = lost to follow-up; MoH = Ministry of Health; PEPFAR = U.S. President’s Emergency Plan for AIDS Relief

In the following, we present clinician and peer perspectives of the implementation barriers to centralized VLM and opportunities for optimizing routine VLM in Uganda. Next, we describe enabling and constraining factors to implementing PoC VLM in the study area, presenting salient themes related to the perceived feasibility and appropriateness of PoC VLM along the following CFIR domains (see Fig. 1): intervention attributes (technological level), characteristics of individuals (client level), inner setting (organizational level), and outer setting (structural level).

### Tension for change: gaps and opportunities for optimizing routine viral load monitoring implementation

**Fig. 2:**
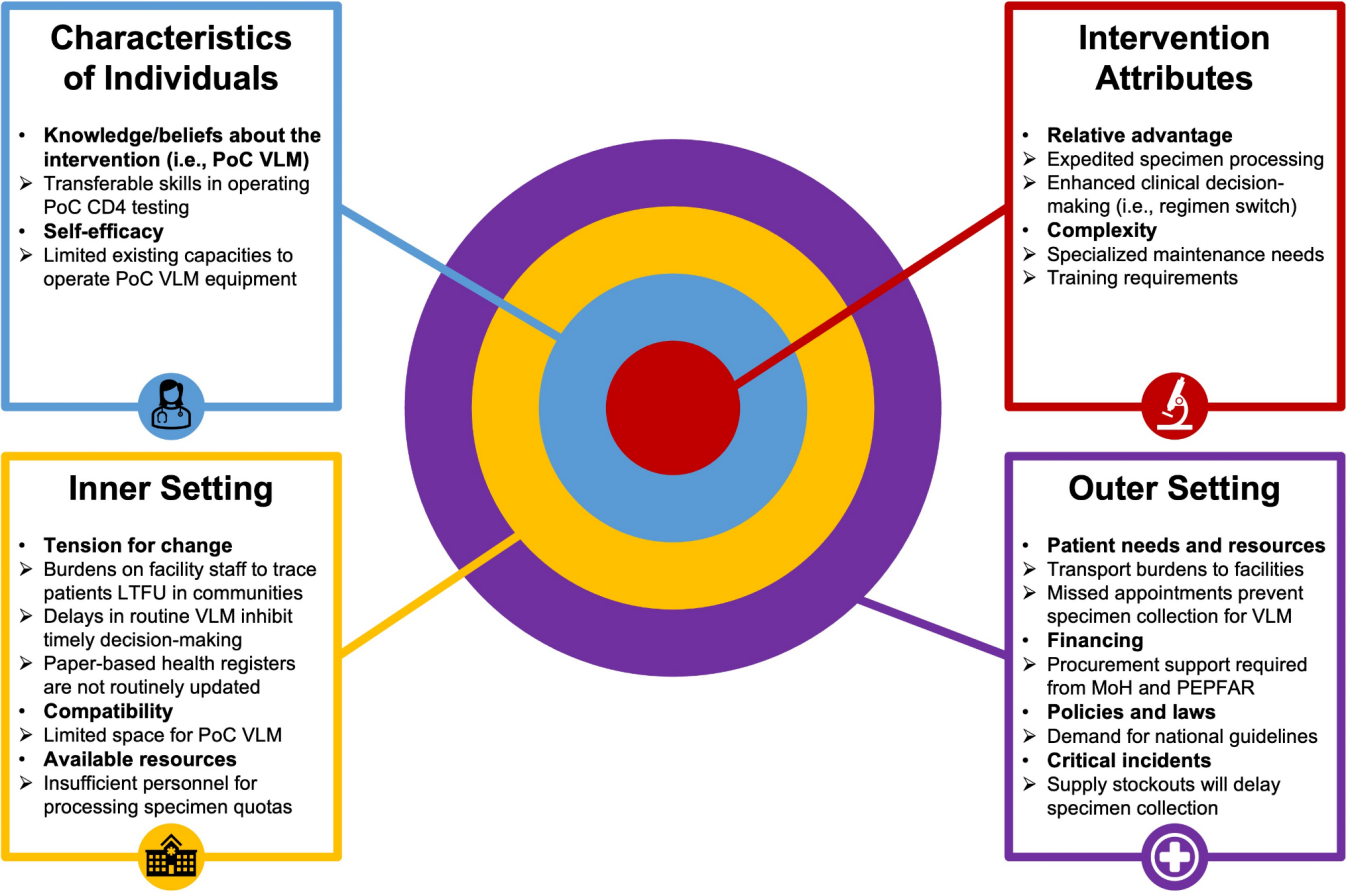
Sequence and provider-reported bottlenecks along the centralized viral load monitoring cascade in Uganda. *Notes*: CPHL = Central Public Health Laboratory; H/C = health center; PCR = polymerase chain reaction; VL = viral load

Clinicians and peer health workers described the current centralized VLM process as one that could manage a high volume of submitted specimens but was highly susceptible to various bottlenecks that could delay specimen processing time. Figure 2 illustrates the various steps and actors engaged in the centralized VLM cascade, beginning with specimen collection at satellite HIV clinics and concluding with viral load results return to clients. At each stage of the centralized VLM cascade, participants identified discrete barriers to specimen custody transfer and processing, including stockouts of medical equipment (e.g., gloves, DBS cards) required for specimen collection, lost/misplaced specimens during transfer to district hubs, CPHL testing backlogs, and resource-intensive client tracing efforts for specimens revealing high-level viremia.

*Sometimes samples…or papers get lost in transit. We bleed [collect a specimen from] the patient*,* but when those things get lost*,* we still assume that results are going to come back. In one or two months*,* we don’t get results*,* so that patient is not ready for another viral load.* –ART In-Charge, Hospital.

*The health worker instructs you to look for that person [client with viremia]…When you reach her home*,* they tell you*,* “She is not around. She went to Kampala.” Or*,* “She went to Tanzania.” Or*,* “She separated from the husband.” When you try calling her*,* the phone is not going through*,* or it is a Tanzanian line…You find it very hard to get those people.* –Peer Health Worker, Health Center IV.

While clinicians reported average specimen processing times of 4–6 weeks, these additional bottlenecks could delay results return for up to 3 months.

### Perceived benefits of point-of-care testing platforms as alternatives to centralized viral load monitoring

Reflecting on salient challenges to centralized VLM, participants responded enthusiastically to the prospect of PoC technology, anticipating accelerated turnaround times in specimen processing as well as shorter and/or fewer required client encounters with HIV care services. Clinicians, in particular, emphasized the promise of expedited clinical decision-making as the source of their enthusiasm for PoC VLM, particularly in a context where delayed specimen processing times were common.

*It will improve turnaround time*,* which will improve decision-making…Sometimes we’re late to make decisions because we do not have the results.* –Nurse, Health Center IV.

*We will be able to monitor our clients in real time. The bureaucracy of sending samples then waiting for results…all of that is broken.* –Clinical Officer, Health Center IV.

Providers also anticipated secondary benefits from PoC VLM implementation to clients themselves, specifically improvements to their quality of care. Participants alluded to the demanding viral load monitoring schedules for clients exhibiting viremia, who in some cases traveled long distances for frequent clinical visits—increasing their likelihood of subsequent disengagement and attrition from HIV care. PoC VLM could, thus, reduce clinical visit burdens for these clients by integrating specimen collection and viral load results return into a single clinical encounter, improving the standard of care.

*Our clients are staying very far [away from the facility]…Instead of waiting for the results*,* we avoid that process of calling them back [to the facility].* –Counselor, Health Center III.

*When they [clients] come to the facility*,* they want to be attended to within the very first minutes of arriving. When they show up on time*,* they do not want to wait long at the clinic.* –Peer Health Worker, Health Center II.

One clinician even anticipated PoC VLM could positively reinforce clients’ HIV self-management practices outside the clinic – specifically daily treatment adherence.

*Even for those who are [virally] suppressed*,* it would be valuable. The results come early*,* and you give recommendations or compliments to the person. And these compliments do a lot…The person gets some positive energy in taking the drugs.* –Nurse, Health Center II.

Lastly, participants predicted that PoC VLM could optimize downstream efficiencies in ancillary clinical management practices. One clinician described how PoC VLM could facilitate appropriate clinical action by quickly distinguishing clients returning to care after receiving treatment from another facility (“silent transfers”) and clients returning to care following a period of clinical attrition (“return to care”), both of whom might otherwise be treated as clinically disengaged populations (“lost to follow-up”).

*It will be good for clients who have disengaged from care…Some of them say they have been taking drugs from another facility. If you can do that viral load*,* you will be able to know whether that client has been on ART or not.* –ART In-Charge, Hospital.

Another clinician described PoC VLM as a conduit to improved health records management, enabling providers to document viral load outcomes in the same clinical encounter as the specimen collection.

*If you collect the sample*,* then immediately get the results*,* you fill in the register*,* and everything is done…If results come in two weeks late…you forget to fill in the register. You have a lot of other things to do.* –Nurse, Health Center II.

### Anticipated barriers and constraints to tapping the full potential of point-of-care viral load monitoring

Despite their enthusiasm for PoC VLM rollout, HIV clinicians and peer health workers articulated constraints across CFIR domains to PoC VLM implementation in their respective practice contexts. At the technological level, participants expressed concerns about the complexity of the PoC instruments and the resources required to sustain their implementation. One nurse at a level-II health center, for example, worried about the “learning curve” of PoC testing instruments and the infeasibility of requiring “people to become testing experts” in his clinical practice. Reflecting upon their experiences with PoC platforms for CD4 cell count monitoring, other clinicians anticipated maintenance requirements of the PoC instruments that would be presently unsupported in their clinics, even in better-resourced, higher-tier facilities.

*These CD4 machines normally break down*,* and we don’t have someone here to repair them. That could be a potential challenge.* –ART In-Charge, Hospital.

Given prominent challenges in the study area with HIV service disengagement and care attrition, driven principally by erratic internal and cross-border mobility, participants also questioned the effectiveness of PoC, relative to centralized, VLM implementation in circumstances when clients missed clinical appointments and, thus, would not submit clinical specimens required for VLM. At one facility where tracing clients with missed appointments required a substantial amount of financial and human resources, the nurse doubted that the benefits of a PoC platform would outweigh its costs, explaining how decentralization would not expedite the VLM efficiencies among clients for whom collecting specimens on the VLM schedule was infeasible.

*The other challenge would be clients not turning up. How are we going to work up on that? If they miss [appointments]*,* that means you cannot do the [viral load] monitoring*. –Nurse, Health Center IV.

Provider-identified barriers to PoC VLM implementation primarily clustered at the organizational level (inner setting), specifically workforce requirements and institutional capacities to effectively manage a decentralized VLM system. Across facility tiers, clinicians worried that the volume of viral load specimens drawn for routine VLM would quickly overwhelm a decentralized VLM system, primarily due to insufficient personnel to collect and process specimens onsite.

*In one month*,* we perform around 1*,*300-1*,*600 viral loads. The human resource burden needs to be considered.* –ART In-Charge, Hospital.

Clinicians further relayed concerns about space requirements to operate a PoC machinery, especially in lower-tier facilities—where space for additional laboratory equipment was already limited.

*We need the space itself for the laboratory. We need facilities to carry out viral load testing because we don’t have anything at the moment.* –ART In-Charge, Health Center II.

Lastly, participants described structural barriers (outer setting) that would need to be addressed to enable a successful PoC VLM rollout. Clinicians explained that without formal approval and sanctioning by the Ministry of Health, specifically integration of PoC VLM into its national HIV treatment guidelines, expanded decentralization of VLM would remain a distant aspiration rather than a proximal reality. Beyond protocols, overcoming chronic supply shortages in the VLM pipeline would be a prerequisite to making PoC VLM the standard of care.

*We sometimes run short of supplies*,* especially gloves. The lab team cannot work without gloves.* –Clinical Officer, Health Center IV.

## Discussion

Our qualitative inquiry into the perceived feasibility and appropriateness of PoC VLM in rural Uganda identified both opportunities to strengthen the performance of the existing centralized VLM system and practical constraints to successful implementation of decentralized VLM. These anticipated implementation constraints, however, did not diminish clinician or peer health worker enthusiasm for the numerous perceived advantages of PoC VLM, particularly its potential to accelerate viral load results return for timely clinical action (e.g., referral to ART adherence counseling, drug resistance testing). Participants also described secondary operational benefits of PoC VLM implementation, from reduced demands/costs of tracing harder-to-reach clients exhibiting viremia to increased precision in triaging individuals returning to HIV care following periods of disengagement. In a context where substantial mobility [28, 36], “silent” or self-transfers between HIV clinics [23, 37], and informal ART borrowing/sharing [28, 38] pose challenges to long-term retention in HIV services, PoC VLM can optimize efficiencies in HIV treatment provision and clinical management of complex, intermittently care-engaged clients.

Identified prospective benefits of PoC VLM further underscore the technology’s potential to increase client satisfaction with their HIV care, which is a precursor to sustained engagement with HIV services [39–41]. Participants hypothesized that PoC VLM would improve client perceptions of HIV care quality through various pathways, including reduced transportation demands and clinical visit burdens for clients—facilitated by viral load results return in the same clinical encounter as specimen collection—as well as positive reinforcement from clinicians to maintain ART adherence. In Uganda, like other sub-Saharan African countries, viral load suppression or undetectability identified during routine VLM is seldom communicated to clients outside of infrequent (6- or 12-month intervals) HIV care appointments, representing a missed opportunity to communicate clinical outcomes to clients in a manner that reinforces their HIV self-management practices [42–45]. Beyond enhanced clinical management of clients exhibiting viremia, PoC VLM has potential to increase the quality of clinical visits for clients stable on ART, who tend to have less frequent contact with the HIV care system relative to clients experiencing ART adherence challenges and/or virologic failure.

Nevertheless, provider-articulated constraints to VLM decentralization primarily clustered around institutional resource requirements for sustainable implementation. Salient barriers to PoC VLM implementation included installation and maintenance costs of PoC testing platforms, inadequate physical space and laboratory capacity to operate a PoC VLM system, and insufficient personnel for processing the volume of specimens collected for routine VLM. These prospectively identified challenges are comparable to lessons learned from the scale-up of other PoC technologies in African HIV treatment programs, specifically CD4 cell count monitoring platforms [46, 47]. Additional research is warranted into feasible PoC VLM implementation strategies that capitalize upon its promise of expedited viral load results return without overwhelming local health system capacities, especially in under-resourced satellite HIV clinics.

Our findings must be considered with a few limitations in mind. First, our study formatively assessed appropriateness and feasibility of PoC VLM in rural Uganda. While participants reflected on their experiences with other PoC testing platforms (i.e., for CD4 cell count monitoring), most participating clinicians communicated no prior experiences with PoC VLM implementation; perspectives of a hypothetical implementation strategy like decentralized VLM may not align with actual implementation experiences with PoC VLM. Second, interviews and focus groups with HIV clinicians and peer health workers uncovered provider perceptions of PoC VLM’s feasibility and appropriateness, but we did not purposively elicit perspectives of the technology from persons living with HIV outside the health workforce, whose perspectives may diverge from those of service providers. Finally, although our results complement and build upon the bourgeoning—albeit scant—literature on PoC VLM implementation experiences in sub-Saharan Africa, our findings may not necessarily transfer to clinical settings outside of rural south-central Uganda.

## Conclusions

Taken together, findings from qualitative interviews and focus groups with HIV clinicians and peer health workers in rural Uganda affirmed the appropriateness of PoC VLM to address bottlenecks along the routine VLM cascade, which were perceived to delay clinical action and impede quality clinical monitoring of people living with HIV. However, we also identified implementation constraints at multiple ecological levels that could render PoC testing platforms for routine VLM infeasible in this setting. To ensure a successful and sustainable PoC VLM rollout, PoC testing platforms should be prioritized for introduction in facilities with pre-existing resources and capabilities for implementation. Additionally, differentiating PoC VLM eligibility in the early stages of rollout—for example, prioritizing testing for clients who could benefit most from immediate clinical action (e.g., individuals returning to care, clients with histories of ART adherence challenges or recently switched to new ART regimens)—could accommodate local health system capacity to implement decentralized VLM.

## Data Availability

The datasets used and/or analyzed during the current study are available from the corresponding author on reasonable request.

## Abbreviations

ART: Antiretroviral therapy
CFIR: Consolidated Framework for Implementation Research
CPHL: Central Public Health Laboratory
DBS: Dried blood spots
PoC: Point-of-care
VLM: Viral load monitoring
